# Fabrication of Magnetic-Antimicrobial-Fluorescent Multifunctional Hybrid Microspheres and Their Properties

**DOI:** 10.3390/ijms14047391

**Published:** 2013-04-02

**Authors:** Ling-Han Xiao, Tao Wang, Tian-Yi Zhao, Xin Zheng, Li-Ying Sun, Ping Li, Feng-Qi Liu, Ge Gao, Alideertu Dong

**Affiliations:** 1 College of Chemistry and MacDiarmid Laboratory, Jilin University, Changchun 130021, China; E-Mails: xiaolinghan1981@163.com (L.-H.X.); luobo85@yahoo.com.cn (T.W.); tianyi63@yahoo.com.cn (T.-Y.Z.); zhengxin3699@yahoo.cn (X.Z.); bumixiu@126.com (L.-Y.S.); lipinghuaxue19@gmail.com (P.L.); liufengqi@jlu.edu.cn (F.-Q.L.); 2 College of Chemistry and Chemical Engineering, Inner Mongolia University, Hohhot 010021, China

**Keywords:** poly(glycidyl methacrylate), magnetic, antibacterial, fluorescent, multifunctional, hybrid microspheres

## Abstract

Novel magnetic-antimicrobial-fluorescent multifunctional hybrid microspheres with well-defined nanostructure were synthesized by the aid of a poly(glycidyl methacrylate) (PGMA) template. The hybrid microspheres were fully characterized by scanning electron microscopy (SEM), transmission electron microscopy (TEM), Fourier transform infrared (FTIR), X-ray diffraction (XRD) and digital fluorescence microscope. The as-synthesized microspheres PGMA, amino-modified PGMA (NH_2_-PGMA) and magnetic PGMA (M-PGMA) have a spherical shape with a smooth surface and fine monodispersity. M-PGMA microspheres are super-paramagnetic, and their saturated magnetic field is 4.608 emu·g^−1^, which made M-PGMA efficiently separable from aqueous solution by an external magnetic field. After poly(haxemethylene guanidine hydrochloride) (PHGH) functionalization, the resultant microspheres exhibit excellent antibacterial performance against both Gram-positive and Gram-negative bacteria. The fluorescence feature originating from the quantum dot CdTe endowed the hybrid microspheres with biological functions, such as targeted localization and biological monitoring functions. Combination of magnetism, antibiosis and fluorescence into one single hybrid microsphere opens up the possibility of the extensive study of multifunctional materials and widens the potential applications.

## 1. Introduction

Guanidinium antibacterial materials have recently attracted considerable scientific attention, due to their unique advantages, such as high antibacterial activity, high stability, ease of storage, reproducibility, non-corrosiveness, non-toxicity and low cost. They have been widely utilized in various fields, e.g., medical devices, hospitals, water purification systems, food packaging, food preservation and sanitation [1–8]. As for a contact biocide, the contact area of guanidinium materials with microorganisms is very crucial to their antibacterial efficiency. Reduction in the size of the guanidinium antibacterial materials can increase their specific surface area and provide more effective antimicrobial functional groups and, thus, can improve the antimicrobial efficiency. Nano-materials have shown huge potentials and good prospects for their application in many fields, due to their outstanding properties, such as small size, large surface area and high reactivity [9]. Thus, increase of the specific surface area via preparing the guanidinium antibacterial materials with a nano-size could be an effective method to improve the antibacterial properties [10].

However, guanidinium antibacterial nano-materials still have some drawbacks, such as the cumbersome recovery process. The antibacterial process is usually carried out in an aqueous solution, and then residual antimicrobial nano-material should be isolated from a large amount of the aqueous solution by an expensive separation procedure. Introduction of a magnetic component into the antibacterial system is a necessary and effective way to reduce the recovery cost, due to its unique properties in the magnetic separation technology [11,12]. Magnetic separation is an important technique, which separates and recovers magnetic particles or particles susceptible to magnetic fields through an external magnetic field [13,14]. Micro/nano-particles and biological particles could be separated from the aqueous solution quickly and easily by utilizing this technology [15–17]. The greatest advantage of this technique is to isolate the maximum target substance in the shortest time. Since the mid-1970s, magnetic separation technology has been widely applied in the bioprocessing and biomedical fields, such as enzyme immobilization, cell arrangement, protein separation and drug delivery [18].

Fluorescent labeling is an important biotechnology, which plays a key role in biological imaging and biological monitoring. With the in-depth study of the fluorescent substance, the scientists found that the quantum dots have many advantages compared to conventional organic fluorescein for good stability, wide excitation wavelength, stable high quantum yield, highly narrow emission peaks and other aspects. As a fluorescent probe, doped quantum dot fluorescent microspheres can play a pivotal role in biological imaging and biological monitoring. The quantum dot CdTe were adsorbed on the polymer surface by electrostatic adsorption, so that the polymer has fluorescence, which has targeted localization and biological monitoring functions [19].

**Figure 1:**
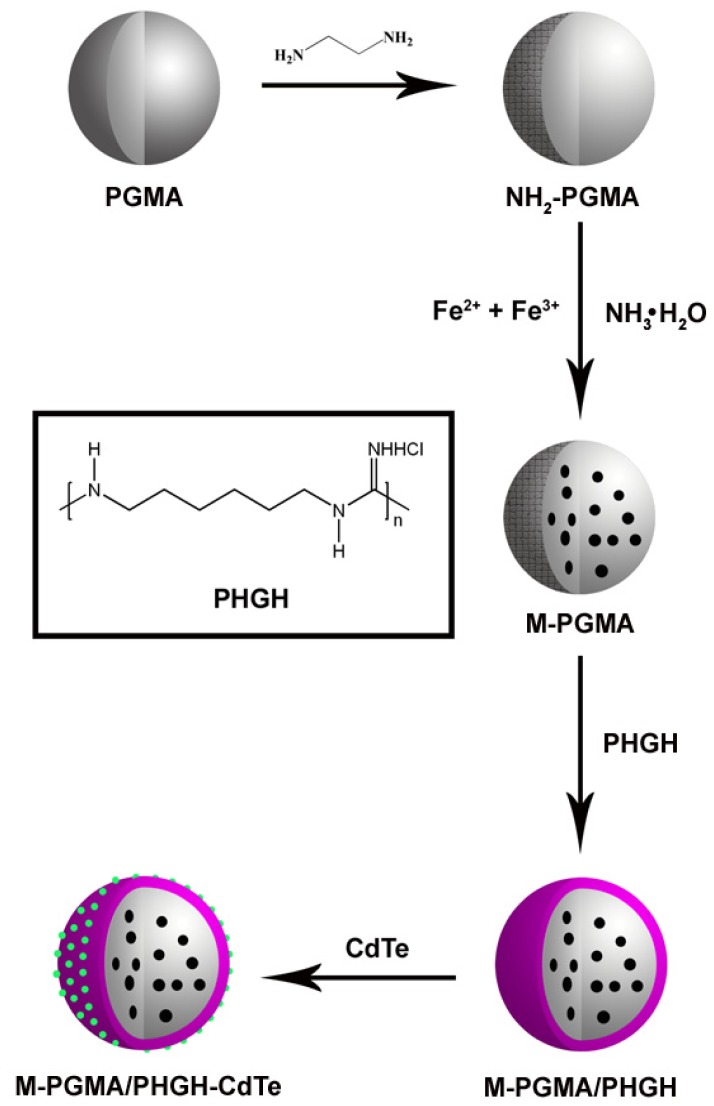
Schematic illustration for the formation processes of the multifunctional M-poly(glycidyl methacrylate) (PGMA)/poly(haxemethylene guanidine hydrochloride) (PHGH)-CdTe hybrid microspheres.

In this paper, poly(glycidyl methacrylate) (PGMA) was chosen as the cladding object, due to its high reactivity caused by superficial epoxy groups and good resistance to acid, heat, weather and solvent. Multifunctional hybrid microspheres with magnetic, antibacterial and fluorescent function were obtained using a PGMA template, as shown in Figure 1. The as-synthesized microspheres could be effectively recovered, due to their magnetic character, and they could be used in several areas, such as biological imaging and biological monitoring, because of their fluorescent property. For an antimicrobial test, *Staphylococcus aureus* (*S. aureus*), *Bacillus subtilis* (*B. subtilis*), *Escherichia coli* (*E. coli*) and *Pseudomonas aeruginosa* (*P. aeruginosa*) were used as model bacteria, and hybrid microspheres exhibited distinguishable antibacterial activity against all of them.

## 2. Results and Discussion

### 2.1. Description

PGMA microspheres capable of responding to magnetic fields were prepared by dispersion polymerization of GMA in the presence of iron oxides, as reported by Daniel Horák, and the conditions for synthesis and properties of the resulting magnetic carriers were studied systematically. Subsequently [20], micron-sized, monodisperse, superparamagnetic, luminescent composite poly(glycidyl methacrylate) (PGMA) microspheres with functional amino-groups were successfully synthesized by Chang *et al.*[21]. The composite microspheres were bright enough, easily observed using a conventional fluorescence microscope and were easily separated from solution by magnetic decantation using a permanent magnet. Although authors claimed that these new multifunctional composite microspheres were promising in a variety of bio-analytical assays involving luminescence detection and magnetic separation, no analytical applications were reported. Soon afterward, Jing *et al.* synthesized multifunctional PGMA microspheres with the capability of recognizing and binding cancer cell by the aid of the magnetic and fluorescent PGMA microspheres as support [22]. Hela cells could be captured with these PGMA microspheres from their suspension and easily moved in the direction of the magnetic field. On the basis of these studies mentioned above, magnetic-fluorescent bifunctional PGMA microspheres herein were further functionalized with antibacterial guanidinium to obtain magnetic-antimicrobial-fluorescent multifunctional microspheres. In this work, the combination of magnetism and fluorescence, with antibacterial performance in one single entity has been utilized for the first time to produce multifunctional nanocomposites.

The multifunctional M-PGMA/PHGH-CdTe microspheres were skillfully fabricated by the encapsulation of magnetic PGMA nanoparticles with antibacterial PHGH and the fluorescent CdTe attached to the PHGH shell. Figure 1 represents vividly the synthetic procedure of the multifunctional M-PGMA/PHGH-CdTe microspheres. Firstly, the PGMA support microspheres were modified by ethylene diamine to yield NH_2_-PGMA microspheres, and the magnetic M-PGMA microspheres are subsequently prepared by *in situ* precipitation. The PHGH coated magnetic PGMA nanoparticles were obtained by the aid of epoxy chloropropane (ECH) as mediator. At last, the fluorescent quantum dot CdTe were attached to the outer PHGH shell by electrostatic adsorption to obtain microspheres. Introducing magnetic PGMA nanoparticles as templates for preparation of PHGH shell enhances the materials’ surface area and provides an effective means for the separation of the antibacterial agents through the application of a magnetic field. In addition, the quantum dot fluorescent microspheres can play a pivotal role in biological imaging and biological monitoring as a fluorescent probe. Similar strategies can also be utilized for the fabrication and separation of other multifunctional materials.

### 2.2. SEM Test of the PGMA, NH_2_-PGMA and M-PGMA Microspheres

**Figure 2:**
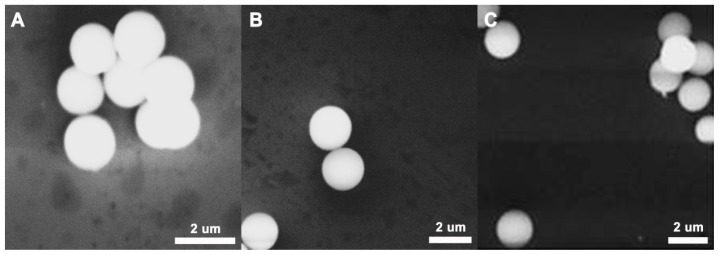
SEM images of hybrid microspheres: (**A**) PGMA; (**B**) NH_2_-PGMA; (**C**) M-PGMA.

SEM was carried out to investigate the morphology, structure, surface state and particle size of the as-synthesized microspheres. Figure 2A–C present the representative SEM images of the prepared PGMA, NH_2_-PGMA and M-PGMA microspheres. PGMA, NH_2_-PGMA and M-PGMA microspheres are quasi-monodisperse, spherical, surficial smooth and solid, and the mean particle size is 1.64, 1.77 and 1.81 μm, respectively. The increase in particle size further implied the successful formation of each synthetic step. However, there is no significant difference in particle surface between NH_2_-PGMA and M-PGMA microspheres, suggesting that Fe_3_O_4_ nanoparticles prepared by *in situ* deposition were mainly deposited inside of the microspheres.

### 2.3. Characterization of the M-PGMA/PHGH-CdTe Microspheres

**Figure 3:**
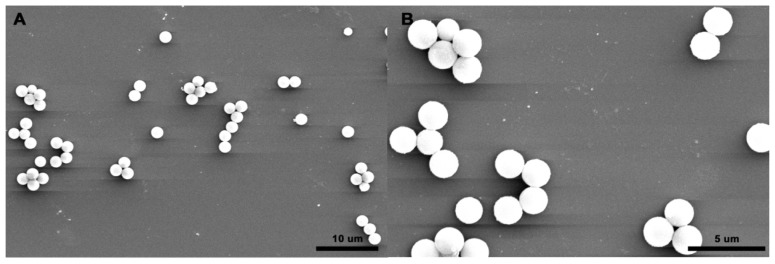

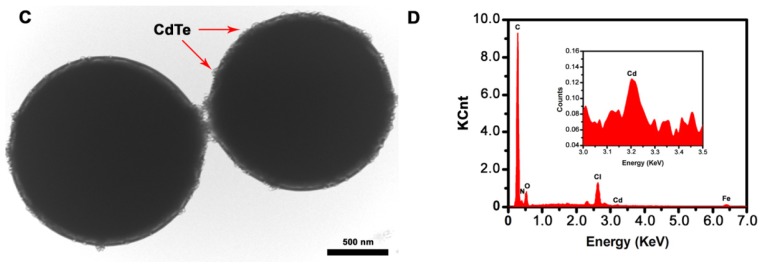
(**A**,**B**) TEM images of M-PGMA/PHGH-CdTe microspheres; (**C**) SEM images and (**D**) EDX spectrum of the M-PGMA/PHGH-CdTe microspheres.

Figure 3A,B presents the representative SEM images of the prepared *M-PGMA/PHGH-CdTe* microspheres. The microspheres are quasi-monodisperse, spherical, surficial smooth and solid, and the mean particle size is 2.0 μm. Figure 3C depicts TEM images of the M-PGMA/PHGH-CdTe microspheres. In Figure 3C, the typical core–shell structure of PHGH with CdTe coated magnetic PGMA microspheres can be discerned. The M-PGMA/PHGH-CdTe microspheres are obviously spherical in shape, and the modified iron oxide nanoparticles are in the center of the microspheres. It can be seen that the quantum dots CdTe, which the arrow annotations denote in Figure 3C, were almost spherical attached to the PHGH shell, as indicated in Figure 1. The compositional information of the EDX spectrum in Figure 3D exhibits the presence of C, N, O, Cl, Fe and Cd elements in the M-PGMA/PHGH-CdTe microspheres. The signal of the Fe element is readily detected from Figure 3D, providing powerful evidence that the Fe_3_O_4_ has successfully deposited in PGMA microspheres. It is observed clearly for the tiny content of Cd coming from the quantum dot CdTe in the enlarged figure (inset, Figure 3D). The N and Cl peaks appearance confirm the formation of PHGH copolymer on the surface of the PGMA microspheres.

### 2.4. FT-IR Analysis of PGMA, NH_2_-PGMA and M-PGMA Microspheres

**Figure 4:**
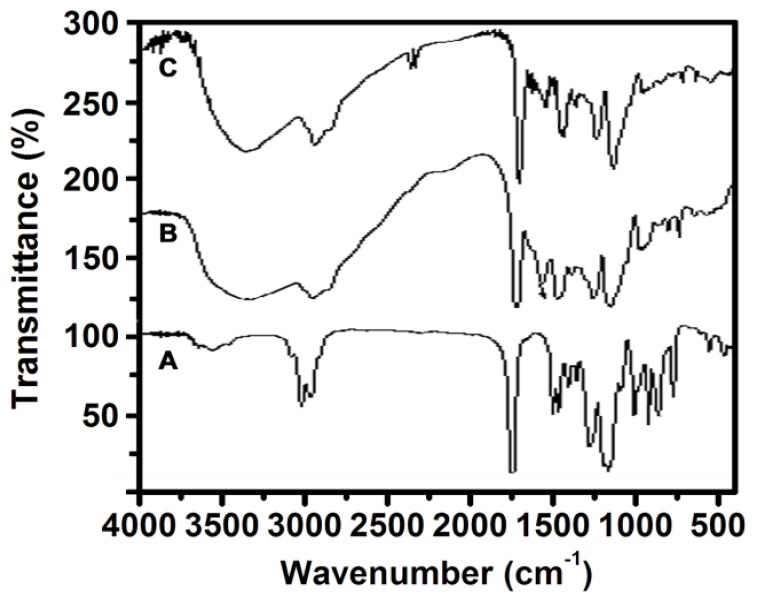
FTIR spectra of (**A**) PGMA; (**B**) NH_2_-PGMA; (**C**) M-PGMA hybrid microspheres.

FTIR spectra were recorded and used to identify the formations of the PGMA, NH_2_-PGMA and M-PGMA microspheres in each synthetic route. As shown in Figure 4A, the strong peaks at 1732 and 1256 cm^−1^ are ascribed to the carbonyl group characteristic absorption and the symmetric stretching vibrations of the epoxy group, respectively [23]. The peaks at 848 and 910 cm^−1^ are assigned to the asymmetric stretching vibration peak of the epoxy group. In Figure 4B, NH_2_-PGMA microspheres have shown the disappearance of the epoxy group-symmetric stretching vibration peaks at around 848 and 910 cm^−1^ compared with the original PGMA, while new peaks appeared at 3310 and 1568 cm^−1^ corresponding to the –NH and –NH_2_ stretching vibration, respectively [24]. These data indicated that the modification with ethylenediamine on the PGMA surface is successfully performed. In Figure 4C, the peak at 580 cm^−1^ is attributed to the Fe-O stretching vibration, indicating that the Fe_3_O_4_ has successfully deposited in PGMA microspheres via *in situ* deposition [23].

### 2.5. XRD Analysis of M-PGMA/PHGH Microspheres

**Figure 5:**
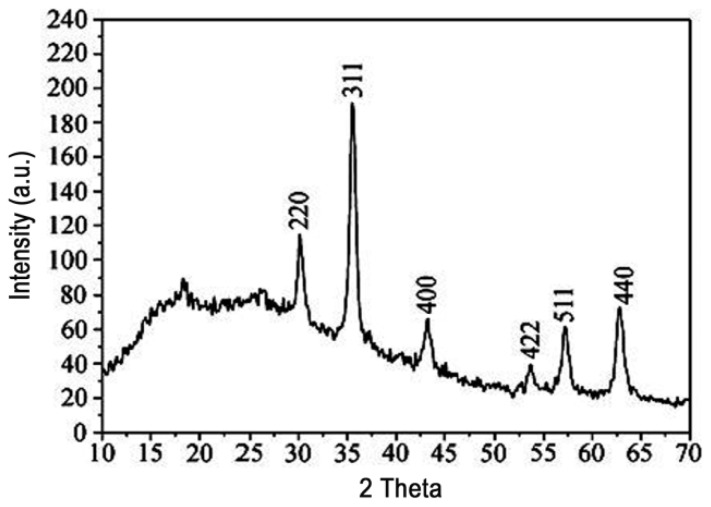
XRD patterns of the M-PGMA/PHGH hybrid microspheres. (a.u. = arbitrary unit).

XRD patterns of the magnetic M-PGMA/PHGH microspheres (Figure 5) show characteristic (220), (311), (400), (422), (511) and (440) peaks of cubic inverse spinel structure, which matched well with standard Fe_3_O_4_ nanoparticles [25]. The pattern of the M-PGMA/PHGH microspheres shows a broad band appearing in the range from 15° to 25°, which is derived from the amorphous PGMA component and polymer shell [26]. No other impurities were observed on XRD patterns, suggesting that the as-synthesized microspheres were relatively pure. It is obvious that the characteristic diffraction peaks of Fe_3_O_4_ are weakened in the pattern of the magnetic M-PGMA/PHGH microspheres, because of the presence of the amorphous template. From XRD results, it can be concluded that the iron oxide nanoparticles are encapsulated by PGMA, which is in good agreement with the SEM and TEM results.

### 2.6. Magnetism Assessment of M-PGMA/PHGH-CdTe Microspheres

**Figure 6:**
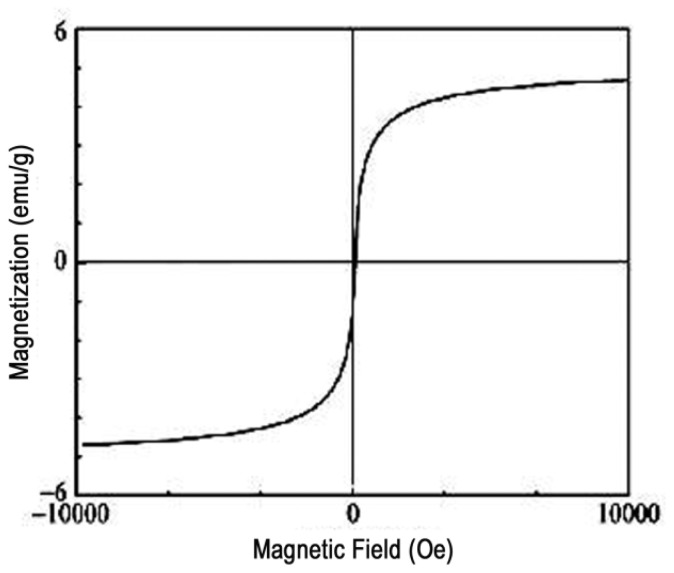
The magnetic hysteresis loop of the M-PGMA/PHGH-CdTe microspheres at 298 K.

The magnetic hysteresis loop of the M-PGMA/PHGH-CdT*e* hybrid microspheres was taken at room temperature (Figure 6). M-PGMA/PHGH-CdTe hybrid microspheres displayed a super-paramagnetic property, and the saturation magnetization value was found to be 4.608 emu·g^−1^, which is sufficient for magnetic separation from water solution. The saturation magnetization value of the M-PGMA/PHGH-CdTe microspheres is less than that of the pure magnetic iron oxide nanoparticles (61.87 emu·g^−1^), which can be explained by considering the diamagnetic contribution of the PGMA template surrounding the magnetic nanoparticles [27].

**Figure 7:**
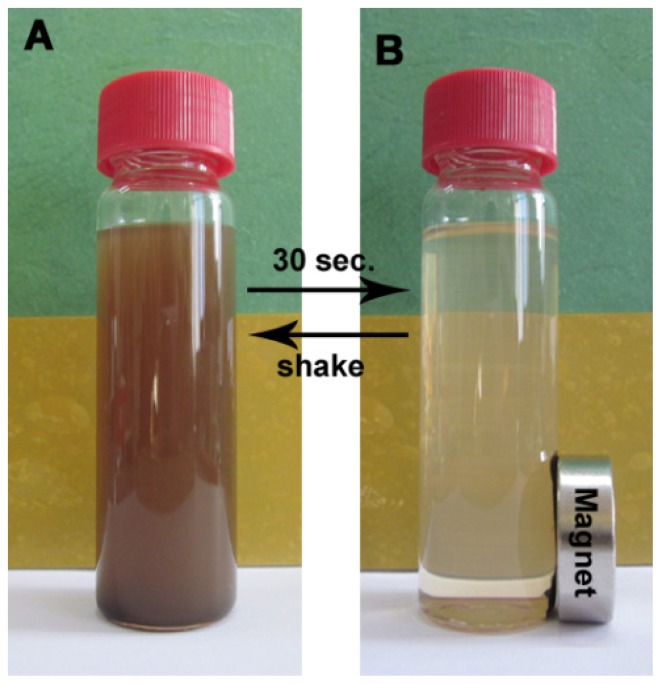
The photograph of the magnetic M-PGMA/PHGH-CdTe microspheres dispersed in aqueous solution without and with an external magnetic field.

M-PGMA/PHGH-CdTe hybrid microspheres were dispersed in water to study their magnetic separation behavior. Figure 7 exhibits that M-PGMA/PHGH-CdTe hybrid microspheres rapidly move toward the magnetic field direction in an aqueous solution under the external magnetic field in 30 s (from Figure 7A to Figure 7B). After the cancellation of the external magnetic field, M-PGMA/PHGH-CdTe hybrid microspheres evenly spread out in water again with bottle shaking (from Figure 7B to Figure 7A). In addition, introducing the magnetic separation can make the M-PGMA/PHGH-CdTe hybrid microspheres recyclable. These microspheres are potent biocidal agents, which can be easily separated and mechanically directed by applying a magnet. This is a utility when a particle-bound bacteria needs to be captured for environmental monitoring or the particles are to be directed specifically to a location of bacterial colonies, such as in a water treatment system and cooling devices and pipes. Thus, the recyclable antibacterial performance of M-PGMA/PHGH-CdTe hybrid microspheres is the crucial point of our study and will be taken into account in our consequent work for their practical application.

### 2.7. The Fluorescence Assessment of M-PGMA/PHGH-CdTe Microspheres

**Figure 8:**
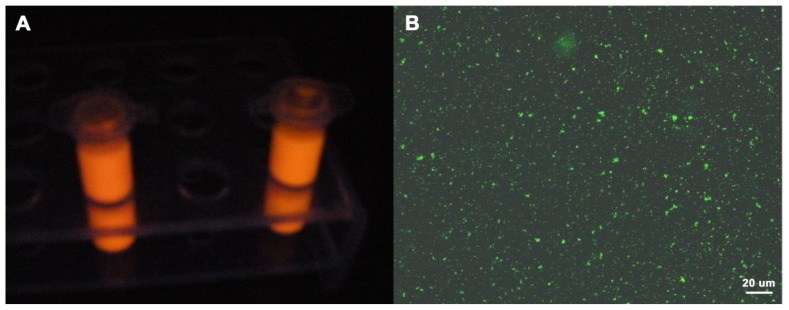
(**A**) The photograph of composite microspheres dispersed in water under ultraviolet irradiation; (**B**) Digital fluorescence microscope image of composite microspheres dispersed in water.

Introducing the quantum dots CdTe can provide the resultant hybrid microspheres fluorescent feature, which was confirmed through fluorescent assessment by dispersing the microspheres into aqueous solution [28]. M-PGMA/PHGH-CdTe hybrid microspheres render orange-red fluorescence under the irradiation of the ultraviolet lamps in Figure 8A and show green fluorescence under the digital fluorescence microscope in Figure 8B. The fluorescent property of resultant hybrid microspheres verified well that the quantum dot CdTe were firmly immobilized on M-PGMA/PHGH hybrid microsphere surface.

### 2.8. Antimicrobial Functions

The antimicrobial property of the resultant hybrid microspheres was examined against *S. aureus*, *B. subtilis*, *P. aeruginosa* and *E. coli* by using the MIC method. MIC is considered to be the lowest concentration that completely inhibits against agar plate comparing, disregarding singly colony or a faint haze caused by the inoculum [29]. The MIC values of M-PGMA/PHGH-CdTe microspheres against *S. aureus*, *B. subtilis*, *P. aeruginosa* and *E. coli* are shown in Table 1. The quantitative data in Table 1 have been repeated three times, and the same results were obtained. Hybrid microspheres have powerful antibacterial property against both Gram-positive bacteria and Gram-negative bacteria and the MIC values of 64 mg/mL, 500 μg/mL, 32 μg/mL and 16 μg/mL against *E. coli*, *P. aeruginosa*, *S. aureus* and *B. subtilis*, respectively.

Guanidine compounds with a broad spectrum of activity against both Gram-positive and Gram-negative bacteria are mainly used as ideal antimicrobial agents. Among various types of guanidine compounds, polymer synthesized from hexamethylene and guanidine salt have been the most extensively investigated, due to high water solubility, wide spectrum antibacterial activity, excellent antibacterial efficiency and nontoxicity. Zhang *et al.* synthesized polyhexamethylene guanidine hydrochloride and polyhexamethylene biguanidine hydrochloride by melting polymerization, and the corresponding antibacterial assessments against bacteria and fungi were carried out as well [6]. Qian *et al.* fabricated modified guanidine polymers composed of PHGH and an epoxy group by using condensation polymerization. The dynamic antimicrobial process of the guanidine polymer and the morphological change of bacterial cells were found by an atomic force microscope (AFM) test [30]. Guan *et al.* grafted guanidine polymer PHGH onto the cellulose fibers by *in situ* graft copolymerization of glycidyl methacrylate-modified PHGH onto cellulose fibers. Through PHGH functionalization, the modified cellulose exhibited high antimicrobial activity against *E. coli*[7]. The hypothesis for this study is that the smaller the functionalized particle is, the more active it will be, for smaller particles possess a larger surface area and, thus, provide more functional sites to contact the bacteria, which results in the improved antibacterial efficiency. To improve the antibacterial effect, M-PGMA/PHGH-CdTe microspheres with a nanostructure were prepared in this work by immobilization of guanidine polymer PHGH into nano-scale M-PGMA, and the antimicrobial activities get a great improvement against both Gram-positive bacteria and Gram-negative bacteria compared with bulk powder counterparts.

## 3. Experimental Section

### 3.1. Materials

Guanidine hydrochloride was purchased from Tianjin Huadong Chemical Reagent Plant. GMA was obtained from Tianjin Guangfu Fine Chemical Research Institute. Ferric chloride (FeCl_3_), potassium hydroxide (KOH), ethylenediamine, Ferrous chloride (FeCl_2_·4H_2_O), epoxy chloropropane (ECH) and ammonium hydroxide (25 wt%) were purchased from Beijing Chemical Company. Azobisisobutyronitrile (AIBN) and polyvinyl pyrrolidone (PVP) were available from Tianjin Chemical Reagent Plant and Shanghai Chemical Reagent Plant, respectively. CdTe were synthesized according to the previous literature [31]. The other reagents were analytical grade and were used without any purification.

### 3.2. Characterization

The morphology and structure of the samples were characterized using a XL30 ESEM-FEG scanning electron microscope (SEM). The XRD patterns were obtained with a Siemens model D5000 diffractometer equipped with a copper anode producing X-rays with a wavelength of 1.5418 Å. Data was collected in continuous scan mode from 10° to 80° with a 0.02° sampling interval. Fourier transform infrared (FTIR) spectra were recorded by using a Thermo Nicolet (Woburn, MA, USA) Avatar 370 FTIR spectrometer. Magnetization curves as a function of magnetic field were measured at 298 K under a magnetic field up to 10 kOe. The fluorescence of the samples was characterized using a NIKON PE2000-u digital fluorescence microscope.

### 3.3. Preparation of PGMA Microspheres

About 0.15 g of PVP was added into a mixed solution containing 120 mL of ethanol and 15 mL of deionized water. The mixed solution was added into a 250 mL flask in a 50 °C water bath after complete dissolution. Then 0.12 g of AIBN was mixed with 5 mL of GMA and subsequently added into the flask; the temperature was increased to 70 °C, and the reaction was kept for 16 h. After the polymerization, the whole system was cooled down, and the obtained microsphere emulsion was centrifuged and washed several times with ethanol and water. At last, the product was freeze-dried and preserved.

### 3.4. Preparation of NH_2_-PGMA Microspheres

About 2 g of the PGMA were added to a mixture of 20 mL of deionized water and 20 mL of ethanediamine. The mixture was stirred vigorously at 80 °C for 12 h. The whole process described above was performed under the protection of N_2_, and the solution was kept stirring at a certain rate. The resultant microspheres were centrifuged and washed several times with ethanol and water, respectively, to remove the impurities and freeze-dried in a vacuum.

### 3.5. Preparation of M-PGMA Microspheres

About 1 g of NH_2_-PGMA was added into 20 mL deionized water and dispersed to emulsion at room temperature. Then, the emulsion was cooled down to 10 °C. FeCl_3_ (162 mg), and FeCl_2_·4H_2_O (63 mg) was mixed in 10 mL of water at 10 °C with stirring. The mixed solution was added into the dispersed NH_2_-PGMA emulsion, and the system was pumped into vacuum rapidly down to a pressure of 10 mm Hg for 20 min. Then, the system was restored to the atmosphere pressure. About 2 mL of NH_3_·H_2_O (25 wt%) was added into the flask, and the temperature was raised to 80 °C. The reaction was kept for 30 min, and the obtained M-PGMA microsphere emulsion was centrifuged several times and dispersed with ethanol and water to remove the impurities and freeze-dried in a vacuum.

### 3.6. Preparation of M-PGMA/PHGH Microspheres

Immobilization of PHGH on M-PGMA microspheres was accomplished via a two-step processes, including epoxide modified PHGH and PHGH immobilization. First, a 5 g mount of PHGH was added into 50 mL of deionized water. ECH (1 g) was added dropwise into the mixture during the last 30 min and kept stirring for 4 h. Then 50 mL of deionized water was added, and the reaction was kept for another 6 h at 60 °C to obtain the epoxide modified PHGH. In the second step, the epoxide-modified PHGH was added into 50 mL of ethanol, and 0.2 g of KOH were added to tune pH = 8. Then, 1 g of M-PGMA was injected into the mixture for 6 h. The obtained M-PGMA/PGHG microspheres were centrifuged and washed several times with ethanol and water, respectively, to remove the impurities and dried in a vacuum oven at 50 °C.

### 3.7. Electrostatic Adsorption of Dot CdTe

Typically, 0.75 mg of M-PGMA/PHGH microspheres were added into a 1.5 mL aqueous CdTe quantum dot solution protected by mercaptosuccinic acid and mixed by sonication for 10 min. After centrifugation, the supernatant was removed, and the precipitate was washed with ethanol, which was dispersed and centrifuged several times to obtain the M-PGMA/PHGH-CdTe microspheres.

### 3.8. Antibacterial Assessment

*Staphylococcus aureus* (*S. aureus*), *Bacillus subtilis* (*B. Subtilis*), *Escherichia coli* (*E. coli*) and *Pseudomonas aeruginosa* (*P. aeruginosa*) were used as model microorganisms to determine the antimicrobial properties of the samples. The minimum inhibition concentration (MIC) of SiO_2_-PS-CDMH nanoparticles was determined by the similar agar plate method. The sample concentration varied from 32, to 64, to 128, to 256, to 512 and to 1024 μg/mL. The culture of each bacterium was diluted by sterile distilled water to *ca*. 100 CFU/mL, and the inoculated plates were incubated at 37 °C for a contact time of 12 h.

## 4. Conclusions

In conclusion, the M-PGMA/PHGH-CdTe hybrid microspheres with the properties of magnetism, fluorescence and antibiosis were successfully synthesized. The magnetic PGMA microspheres were synthesized as support, and PHGH was grafted on the template surface. Finally, the quantum dot CdTe was adsorbed on the microsphere surface. Antibacterial tests revealed that the M-PGMA/PHGH-CdTe microspheres exhibited powerful antibacterial performance against both Gram-positive and Gram-negative bacteria. Magnetic measurement showed that these hybrid microspheres possessed a super-paramagnetic property, and the saturation magnetization value was found to be 4.608 emu·g^−1^. Magnetic behavior can make these antibacterial microspheres structural antibacterial materials separable in a rapid and easy way. The resultant hybrid microspheres with the quantum dot CdTe have targeted positioning and biological monitoring functions. Introducing the magnetism and fluorescence in the antibacterial field in this study opens up the possibility of the extensive study of antibacterial materials, widening their potential applications in medical devices, healthcare products, water purification systems, hospitals, dental office equipment, food packaging, food storage, household sanitation, *etc.*

## Figures and Tables

**Table 1:** MIC Values of M-PGMA/PHGH-CdTe microspheres Against *E. coli*, 25922, *P. aeruginosa*, 27853, *S. aureus*, 25923 and *B. subtilis*, 6633.

Antibacterial Result	Gram-negative		Gram-positive	
	
	*E. coli* ATCC 25922	*P. aeruginosa* ATCC 27853	*S. aureus* ATCC 25923	*B. subtilis* ATCC 6633
MIC (μg/mL)	64	500	32	16
